# Social Support as a Predictor of Community Participation After Stroke

**DOI:** 10.3389/fneur.2019.01013

**Published:** 2019-09-20

**Authors:** Kimberly S. Erler, Virginia Sullivan, Sarah Mckinnon, Rebecca Inzana

**Affiliations:** ^1^Department of Occupational Therapy, MGH Institute of Health Professions, Boston, MA, United States; ^2^Department of Communication Sciences and Disorders, MGH Institute of Health Professions, Boston, MA, United States

**Keywords:** neurological rehabilitation, stroke, social support, social participation, community integration

## Abstract

Participation is a primary goal of neurorehabilitation; however, most individuals post stroke experience significant restrictions in participation as they attempt to resume their everyday roles and routines. Despite this emphasis on participation, there is a paucity of evidence-based interventions for optimizing this outcome and a limited understanding of factors that contribute to poor participation outcomes. Caregiver support at discharge from inpatient rehabilitation positively influences physical and psychological outcomes after stroke but more research is needed to understand the association between social support and participation. This study aimed to examine the independent contribution of perceived social support to participation 3 months post discharge from inpatient stroke rehabilitation. This study was a secondary analysis of the Stroke Recovery in Underserved Populations 2005–2006 data. Participants were adults ≥55 years old, living in the community 3 months post discharge from inpatient rehabilitation for ischemic stroke (*n* = 422). Hierarchical linear regressions were performed. The primary variables of interest were the PAR-PRO Measure of Home and Community Participation and the Duke–University of North Carolina Functional Social Support Questionnaire. Perceived social support at discharge from inpatient rehabilitation for ischemic stroke contributed uniquely to the variance in participation 3 months later (β = 0.396, *P* < 0.001) after controlling for race, sex, age, years of education, comorbidities, stroke symptoms, depression, FIM Motor, and FIM Cognitive. Social support accounted for 12.2% of the variance in participation and was the strongest predictor of participation relative to the other independently significant predictors in the model including FIM Motor and depression. There is already a focus on caregiver training during inpatient rehabilitation related to basic self-care, transfers, and medical management. These findings suggest the need for rehabilitation professionals to also address social support during discharge planning in the context of promoting participation. Given the findings, expanding caregiver training is necessary but novel interventions and programs must be carefully developed to avoid increasing caregiver burden.

## Introduction

Stroke is the leading cause of disability in the United States with almost 800,000 people experiencing a new or recurrent stroke each year (1). Stroke can result in a complex matrix of physical, communication, cognitive, and emotional impairments that limit a person's ability to perform basic activities of daily living or participate in the community. The World Health Organization (WHO) International Classification of Functioning, Disability and Health (ICF) provides a scientific basis for examining disability and functioning in the context of a health condition (2). The ICF describes three domains impacted by a health condition: body structures and functions (impairments) which capture functioning at the level of the body, activities which capture functioning at the level of the individual, and participation which captures functioning at the societal level.

Neurorehabilitation aims to minimize disability and restore function after stroke, primarily focusing on the restitution of impairments and compensation for activities of daily living (3). Many rehabilitation professionals expect that an impairment-based or activity-based approach will lead to improvements in community participation (4); however, research indicates that this secondary gain in participation may not always occur. The majority of stroke survivors experience restrictions in participation as they attempt to resume their everyday lives (5–8). Determining modifiable factors that contribute to successful community participation after stroke has the potential to advance clinical practice by informing novel interventions and program development.

Participation is a valued outcome post stroke and often used as a metric of successful rehabilitation (9–12). Participation occurs at the intersection between the person, the activity, and the environment (13, 14). Obembe and Eng (15) suggest that recovery from stroke should be considered successful if the individual resumes the level of community participation similar to his pre-stroke baseline. In samples of healthy adults and adults with chronic conditions, participation has been linked to better overall health and well-being (16–19). Previous research has shown that poor participation after stroke is associated with increased older age (6), worse stroke severity (20), worse physical function (21), worse cognition (22), more comorbidities (21), and increased rates of depression (7); however, the directionality of these relationships remain unknown.

Social support is an environmental factor that has been shown to be a positive prognostic indicator for physical and psychological outcomes after stroke (23–27). Social support can come from a wide range of sources such as family, friends, significant others, social networks, religious organizations, or community groups. Social support can also be the actual assistance a person receives from others or the perceived support that results from the confidence of the availability of support for physical or emotional needs. There is limited evidence to suggest the existence of a positive relationship between social support and participation; however, this relationship needs to be further elucidated. The relationship between perceived social support and participation is particularly important to understand since it is a potentially modifiable factor. A recent systematic review (28) that aimed to determine the relationship between social support and participation post stroke yielded only six articles that met the inclusion criteria, and only one article that included participants in the United States. Norlander et al. (29) found that in addition to driving status and walking distance, the extent of one's social network at 16 months after the first stroke was predictive of more frequent social and leisure activities. Further studies (30, 31) found that both the quality and quantity of social support is associated with participation, but that functional limitations were more strongly related to participation.

Participation is a valued outcome after stroke, and social support has been shown to positively impact other meaningful outcomes. Given the limited evidence in general, and the cultural and health system differences amongst countries, there is a need to better understand the association between participation and the perceived social support after stroke in a sample representative of the United States. The availability of a dataset with a large sample size and the primary predictor and outcome variables of interest as well as many other important confounding variables provides the unique opportunity to test our hypothesis about the relationship between perceived social support and participation to determine feasibility for future prospective research in this area. Hence, the purpose of this study was to determine the unique contribution of perceived social support at discharge from inpatient rehab to participation at 3 months post discharge among individuals with first time ischemic stroke. We hypothesized that, after controlling for other demographic, health, and functional factors, perceived social support at discharge from inpatient rehabilitation would be associated with better participation 3 months later among community dwelling adults with for first time ischemic stroke.

## Methods

### Participants

This study was a secondary analysis of the publicly available dataset, Stroke Recovery in Underserved Populations (SRUP) 2005–2006 database (32). SRUP was an observational cohort study of individuals with first time stroke who received inpatient rehabilitation at one of 11 rehabilitation hospitals in various regions of the country including New Jersey, New York (2), Iowa, California, Illinois, Texas (2), Washington D.C., Kentucky, and Florida. Inclusion criteria required participants to have a diagnosis of first time stroke, be ≥55 years old, and demonstrate the ability to respond to basic questions about orientation. After consent to participate in the study, nursing staff collected demographic and clinical information within 72 h of discharge from the rehabilitation facilities, and trained nurse researchers collected follow up data via telephone at 3 months post-discharge. Further inclusion criteria for our study required participants to have a diagnosis of ischemic stroke (vs. hemorrhagic stroke), be living in a community setting (vs. institutional setting) at the 3-months follow up, and have complete participation and social support data.

### Instruments

Age, sex, race, years of education, number of comorbidities (including arthritis, cancer, respiratory disease, diabetes, heart disease, other circulatory diseases, kidney disease, obesity, mental health diagnoses, or fractures), and number of stroke symptoms (including hemiplegia or hemiparesis, speech difficulties, swallowing difficulties, or neuromuscular symptoms) were derived from demographic and stroke characteristic variables in the SRUP database. Participation, social support, depression, physical function, and cognitive function were assessed with the following measures.

#### Participation

The primary outcome of interest, community participation, was measured with the PAR-PRO Measure of Home and Community Participation (33). The PAR-PRO was developed to complement the FIM, measuring more complex areas of performance (33). Participants were asked the frequency of participation on four items in the domains of socializing inside the home, socializing outside of the home, recreation and leisure activities, and religious or spiritual activities. Response options included no participation in the past month, 1–2 times in the past month, several times in the past month, every week over the past month, and more than once per week. Summary PAR-PRO scores ranged from 0 to 16 with higher scores indicating more community participation. The PAR-PRO has been shown to have good psychometric properties in populations with neurological impairments, including individuals with stroke (33). The PAR-PRO was administered via telephone at 3 months post discharge from inpatient stroke rehabilitation.

#### Social Support

The 11-item Duke–University of North Carolina Functional Social Support Questionnaire (DUFSS) is a measure of perceived social support (34, 35). The DUFSS consists of 11 items (e.g., I have people who care what happens to me) that are scored on a five-point Likert scale with responses ranging from “as much as I would like” to “much less than I would like.” Summary scores range from 11 to 55 with a higher score indicating higher perceived social support. The measure includes items that This DUFSS score was assessed at discharge from inpatient rehabilitation.

#### Depression

The Center for Epidemiologic Studies Depression Scale (CES-D), a 20-item scale with response options ranging from 0 (none of the time) to 3 (most of the time), measured depression (36). Item scores are summed for a total score ranging from 0 to 60 with higher scores indicating more depressive symptoms. This study followed previously established criteria to dichotomize those with and without clinically significant depression using a cutoff score of ≥16 (36, 37). Depression was assessed in person at discharge from inpatient rehabilitation.

#### Physical and Cognitive Function

The FIM Motor and FIM Cognitive subscales measured physical and cognitive disability, respectively (38). The FIM cognitive has five items, and the FIM Motor has 13 items, each rated on a seven-point scale ranging from total assistance (1) to complete independence (7). Higher scores indicate better cognitive and physical function. The FIM Motor and FIM Cognitive were assessed at discharge from inpatient rehabilitation.

### Statistical Analyses

All analyses were performed in IBM SPSS Statistics 25.0 for Windows (39). We conducted hierarchical linear regressions for the dependent variable of participation (PAR-PRO) scores at 3 months post discharge from inpatient rehabilitation for ischemic stroke. Model 1 included the predictors of race, sex, age, years of education, number of comorbidities, number of stroke symptoms, depression, FIM Motor, and FIM Cognitive. Model 2 included all of the predictors in the first model with the addition of social support (DUFSS) scores at discharge from inpatient rehabilitation. The *R*^2^ change between the two models represents the unique contribution of social support to participation after controlling for all other variables in the model. Descriptive statistics were examined for all variables, and model diagnostics (i.e., variance inflation factor and tolerance) were assessed to determine good model fit. A Spearman's Rho Correlation matrix was run to examine the relationship between all variables in the models.

The Institutional Review Board of Partners HealthCare, the Partners Human Research Committee, determined that this research does not meet the definition of human subjects research since investigators performed secondary analyses of an anonymized and publicly available data set, and did not obtain data through an intervention or interaction with individual subjects or identifiable private information about living individuals.

## Results

The original publicly available dataset included 1,219 participants, 891 participants of whom had a primary diagnosis of ischemic stroke (vs. hemorrhagic). Of those with ischemic stroke, 699 lived in a community setting at 3 months post discharge. There were 442 participants who had complete participation data and 422 of those participants also had complete social support data. There were no statistically significant differences on key variables (i.e., age, years of education, number of comorbidities, number of stroke symptoms, FIM Motor, FIM cognitive, sex, race, or depression) between the included 422 and the excluded 277 who had ischemic stroke and were living in the community 3 months post discharge but did not have complete data. Table 1 summarizes the demographics and characteristics of the study sample (*n* = 422). Included participants were admitted to inpatient rehab between December 2005 and October 2006. The relationship between all predictor and outcome variables is depicted in a correlation matrix in Table 2.

**Table 1:** Demographics and characteristics.

**N (%) or mean (*SD*)**	***N* = 422**
Age	68.35 (13.173)
Years of education	12.15 (3.143)
Sex (female)	206 (48.8)
Race (white)	302 (71.6)
Depression status (depressed)	112 (26.5)
Number of comorbidities	2.89 (1.309)
Number of stroke symptoms	1.37 (1.023)
FIM cognitive at discharge	25.94 (6.767)
FIM motor at discharge	60.79 (15.860)
DUKE-UNC FSSQ at discharge	50.33 (7.251)
PAR-PRO at 3 months post discharge	10.443 (4.314)

*SD, Standard deviation*.

**Table 2:** Correlation matrix of all variables.

	**1**	**2**	**3**	**4**	**5**	**6**	**7**	**8**	**9**	**10**	**11**
1. PAR-PRO	–										
2. DUKE-UNC FSSQ	0.511^*^	–									
3. Race	0.081	0.004	–								
4. Sex	−0.033	0.021	−0.075	–							
5. Depression	−0.301^*^	−0.441^*^	0.070	−0.026	–						
6. Age	−0.062	0.092	0.199^*^	0.067	−0.082	–					
7. Years of education	0.068	−0.055	0.108^*^	−0.038	0.017	−0.076	–				
8. # of comorbidities	−0.111^*^	−0.118^*^	−0.030	−0.030	0.064	0.038	−0.095	–			
9. # of Stroke Symptoms	−0.133^*^	−0.107^*^	−0.123^*^	0.019	0.020	−0.091	−0.057	−0.010	–		
10. FIM Motor	0.226^*^	−0.037	−0.016	−0.029	0.076	−0.200^*^	0.047	−0.030	−0.215^*^	–	
11. FIM Cognitive	0.118^*^	−0.029	−0.161^*^	0.023	−0.111^*^	−0.159^*^	0.100^*^	0.005	−0.181^*^	0.492^*^	–

* *Spearman's correlation is significant at the 0.05 level (two-tailed)*.

The base model (Model 1), which included race, sex, age, years of education, number of comorbidities, number of stroke symptoms, depression, FIM Motor, and FIM Cognitive explained 18.0% of the variance in participation at 3 months [*F*_(9, 406)_ = 9.903, *p* < 0.001]. After social support was added to the model (Model 2), all predictors together accounted for 30.2% of the variance in participation at 3 months [*F*_(10, 405)_ = 17.546, *p* < 0.001]. Social support alone accounted for 12.2% of the variance in participation [*F*_(1, 405)_ Δ = 70.977, *p* < 0.001, *R*^2^Δ = 0.122]. In Model 1, number of comorbidities, depression, and FIM Motor had a significant relationship with participation holding all other variables constant. After social support was added in model 2, number of comorbidities was no longer a statistically significant independent predictor of participation. Based on the standardized betas in Model 2, social support (β = 0.396) was the strongest predictor of participation at 3 months relative to the other predictors in the model. Detailed results are in Table 3. Model fit diagnostics indicated an overall good fit for the model. Residuals were fairly normal and homoscedastic in conformance with significance test assumptions, and the variance inflation factor ranged from 1.01 to 1.56, and the tolerance ranged from 0.64 to 0.99, indicating no collinearity among predictors.

**Table 3:** Hierarchical regression results.

**Model 1**			**Model 2**		
	**Standardized** **beta** **(95% CI lower, upper)**	***P*-value**		**Standardized** **beta** **(95% CI lower, upper)**	***P*-value**
Race	0.084 (−0.086, 1.695)	0.077	Race	0.060 (−0.248, 1.40)	0.170
Sex	−0.017 (−0.916, 0.626)	0.711	Sex	−0.016 (−0.848, 0.577)	0.708
Age	−0.047 (−0.046, 0.015)	0.318	Age	−0.073 (−0.053, 0.004)	0.097
Years of education	0.064 (−0.036, 0.213)	0.162	Years of education	0.078 (−0.007, 0.223)	0.066
# of comorbidities	−0.104 (−0.647, −0.046)	0.024	# of comorbidities	−0.052 (−0.453, 0.108)	0.227
# of stroke symptoms	−0.076 (−0.713, 0.068)	0.105	# of stroke symptoms	−0.050 (−0.573, 0.150)	0.251
Depression	−0.292 (−3.753, −1.979)	<0.001	Depression	−0.127 (−2.15, −0.347)	0.007
FIM motor	0.203 (0.025, 0.085)	<0.001	FIM motor	0.243 (0.038, 0.094)	<0.001
FIM cognitive	−0.20 (−0.083, 0.058)	0.728	FIM cognitive	−0.010 (−0.071, 0.059)	0.850
			Social support	0.396 (0.181,0.290)	<0.001
	*R*^2^ = 0.180	<0.001		*R*^2^ = 0.302	<0.001
	$R_{\text{Adj}}^{2}$ = 0.162			$R_{\text{Adj}}^{2}$ = 0.285	
				**R**^2^Δ = 0.122	<0.001

*R^2^, R Square; $R_{Adj}^{2}$, Adjusted R Square; R^2^Δ, R Square Change; R^2^_Adj_Δ, Adjusted R Square Change, CI, Confidence Interval*.

## Discussion

The purpose of this study was to determine the unique contribution of perceived social support at discharge from inpatient rehabilitation for first time ischemic stroke to the participation among community dwelling adults 3 months later. As hypothesized, social support was a highly significant independent predictor of community participation. These findings mirror the evidence for social support and community participation among healthy adults (40) and extend the limited existing literature on social support and community participation post stroke (28).

For first time stroke survivors living in the United States, social support was the strongest predictor of participation among all significant predictors in the model, which included physical function (i.e., FIM Motor) and depression. Social support may impact one's ability to overcome the environmental challenges outside of the home in the setting of a physical impairment and may also act as a protective factor against post stroke depression. This link between social support and community participation highlights the importance of including an individual's social support network in discharge planning from inpatient rehabilitation for ischemic stroke. Although rehabilitation providers include caregiver training in intervention plans, this training typically focuses on activities of daily living, transfers, and medical management without attention to community reintegration and participation (3, 41). Sources of social support, especially perceived social support, can be broad and highly individualized.

While these findings suggest that rehabilitation professionals should train caregivers in strategies to improve social support, it is important to acknowledge the extensive literature on caregiver burden post stroke (42). Caregivers of individuals post stroke who are living at home are charged with new responsibilities that impact their own roles, routines, and ability to resume participation in meaningful activities. Additional training on strategies to optimize perceived social support and community participation may inadvertently increase the burden on caregivers. However, given that social support is an even stronger predictor of community participation than physical function, rehabilitation professionals must develop new approaches for optimizing this outcome without adding further burden to caregivers. Since caregivers are not the only source of perceived social support, rehabilitation professionals should consider working with individuals with stroke to explore other people in their lives who may also provide social support and include them in interventions.

In addition to caregiver burden, access to specialized neurorehabilitation, cost, time, transportation, and post stroke fatigue are a few of the obstacles to delivering time intensive, prolonged in-person interventions that address stroke outcomes across all ICF domains. The growing field of telehealth mitigates many of these challenges by broadening access and maximizing therapy time within the individual's natural environment (43). Telehealth is the provision of healthcare services via telecommunication technology (44). The American Heart Association/American Stroke Association supports the use of telehealth within stroke systems of care for the delivery of occupational therapy, physical therapy, or speech disability assessment and intervention via videoconferencing systems (45). Despite research demonstrating that telerehabilitation post stroke has equal effects compared with conventional rehabilitation and that it may even prevent or minimize the well-documented decline in function that occurs post usual rehabilitation (43, 46), telerehabilitation is not widely implemented. Further, similar to in-person practice, community participation has been overlooked in telerehabilitation with studies primarily examining motor recovery, depression, caregiver burden, and higher cortical dysfunction (46). Telehealth post stroke should include interventions that: (1) provide social support to the individual with stroke, (2) promote interactions with social support networks beyond the caregiver, and (3) address caregiver well-ness. Targeting these areas may improve community participation without additional burden to caregivers or creating challenges for other members of one's social support network.

Strengths of this study include a large sample size of persons with first time stroke from multiple sites across the United States and the use of established outcome measures. Limitations that may affect generalization of these findings to the broader clinical stroke population include the administration of the PAR-PRO via telephone without evidence to support the validity of the scores, the lack of a baseline measure of pre-stroke participation and the lack of measure of satisfaction with participation. It is unknown whether a person had poor participation prior to the stroke or if a person is satisfied with lower levels of participation. Further, this study included participants who were admitted to inpatient rehabilitation for stroke from 2005 to 2006 which may not be a contemporary representation of stroke survivors. Over the last decade, many measures of participation have been developed and studied in the stroke population that may provide a more sophisticated perspective of participation than the PAR-PRO (8). In addition, the dataset did not include time post stroke, so we are unable to make implications beyond the assumption that the individuals were likely admitted to rehab within the first 2 weeks post stroke. There were other potential biases such as non-blinding of the assessors in the original study, and our analysis of a sample only included participants with complete participation and social support data. Lastly, despite the inclusion of demographic and clinical covariates, a large amount of the variance of community participation remains unexplained.

In conclusion, perceived social support at discharge from inpatient rehabilitation for first time ischemic stroke survivors in the United States is the strongest independent predictor of community participation at 3 months post discharge. Although further examination of the unexplained variance is required, it is clear that interventions targeting the outcome of participation should include a social support component without creating additional caregiver burden.

## Data Availability Statement

The Stroke Recovery in Underserved Populations 2005–2006 dataset analyzed for this study can be found in the Archive of Data on Disability to Enable Policy and Research (https://doi.org/10.3886/ICPSR36422.v1).

## Ethics Statement

The Institutional Review Board of Partners HealthCare, the Partners Human Research Committee, determined that this research does not meet the definition of human subjects research since investigators performed secondary analyses of an anonymized and publicly available data set, and did not obtain data through an intervention or interaction with individual subjects or identifiable private information about living individuals.

## Author Contributions

KE, VS, SM, and RI contributed to study concept, drafted the manuscript, and design. KE performed data, statistical analysis, and drafted the tables and figure.

### Conflict of Interest

The authors declare that the research was conducted in the absence of any commercial or financial relationships that could be construed as a potential conflict of interest.
